# Enhancing Research and Development in the Health Sciences as a Strategy to Establish a Knowledge-Based Economy in the State of Kuwait: A Call for Action

**DOI:** 10.3390/healthcare8030264

**Published:** 2020-08-12

**Authors:** Ahmad Salman, Saja A. Fakhraldeen, Sungsoo Chun, Kazi Jamil, Janvier Gasana, Adel Al-Hunayan

**Affiliations:** 1 Ministry of Health, Safat 13001, Kuwait; 2 Environment and Life Sciences Research Center, Kuwait Institute for Scientific Research, Safat 13109, Kuwait; sfakhraldeen@kisr.edu.kw (S.A.F.); kjamil@kisr.edu.kw (K.J.); 3 United Nations Development Programme, Safat 13030, Kuwait; sungsoo.chun@undp.org; 4 Faculty of Public Health, Kuwait University, Safat 13110, Kuwait; Janvier.Gasana@ku.edu.kw; 5 Faculty of Medicine, Kuwait University, Safat 13110, Kuwait; adel.hunayan@ku.edu.kw

**Keywords:** research and development, health sciences, state of Kuwait

## Abstract

Kuwait Vision 2035 is an initiative that was launched in 2017 by His Highness the Emir of the State of Kuwait Sheikh Sabah Al-Ahmad Al-Jaber Al-Sabah. This initiative includes the implementation of a detailed development plan aimed at transforming the state of Kuwait into a regional leader in science, technology, and innovation. Health research will arguably prove to be one of the most impactful research arenas when it comes to accomplishing the goals set forth by the Kuwait Vision 2035 Development Plan. The high impact of health research is derived from its capacity to aid in the establishment of a knowledge-based health industry. The state of Kuwait lacks a system for promoting and managing national R&D efforts. At present, the research and development (R&D) expenditure in the state of Kuwait is far below the international standards that have been shown to lead to innovation and the subsequent development of a knowledge-based economy. Improvement of the weak and unstructured existing R&D apparatus in the State of Kuwait is among the most urgent challenges facing the nation as it strives toward innovation and development of a knowledge-based economy. Developing health research capacities in the State of Kuwait can significantly contribute toward improving public health, health promotion, disease prevention and treatment, and overall human welfare. Importantly, the positive impacts of such extensive benefits will not be restricted to the state of Kuwait and its citizens, but may in fact reap benefits for the global society as a whole. This article first analyzes the current status of healthcare services and health science research in the State of Kuwait, and then summarizes some essential R&D design principles that Kuwait needs to implement in order to achieve the milestones set forth in the Kuwait Vision 2035 Development Plan.

## 1. Introduction

‘High quality healthcare’ is one of the seven pillars forming the foundation of the Kuwait Vision 2035 Development Plan, which is the driving force behind the development efforts set forth by the Kuwait government in 2017 [1]. The state of Kuwait has experienced rapid and extensive economic, socio-demographic, and epidemiological transformations over the past five decades, with an increase in life expectancy from around 50 years to 78 years [2]. Despite this significant improvement in life expectancy, which is indicative of an improvement in healthcare services, the field of healthcare remains in dire need of further significant improvements, particularly in regard to the occurrence of non-communicable diseases (NCDs) [3]. Over the past few decades, the State of Kuwait has witnessed an increase in NCDs, particularly cardiovascular disease, diabetes, asthma, and cancer [3,4]. Overweightness and obesity have reached epidemic proportions in the state of Kuwait, which has the highest rate of adult obesity in the region. Additionally, the prevalence of childhood obesity is alarmingly high in the state of Kuwait and exceeds the prevalence rates reported in neighboring countries and from North America [5,6,7].

Two of the most extensive areas of external reliance in the state of Kuwait are medical technology and the healthcare sector. Healthcare is identified as the most promising field for research and development (R&D) in Kuwait [8]. Despite its immense prospects, the healthcare sector in the state of Kuwait is grossly underdeveloped and is full of weaknesses, both in the context of health knowledge and technical capabilities. Due to the current lack of adequate local medical technology and facilities, cases of overseas treatment for Kuwaiti patients have increased from 3869 cases in 2013 to 16,085 cases in 2017 [9]. The most common cause for sending Kuwaiti patients to receive medical treatment abroad is the occurrence of tumors, of which 16%, 14%, 11%, and 9% are bone, pediatric, gastrointestinal, and cardiac tumors, respectively [9]. Most patients go for treatment to the United Kingdom (30.6%), United States (23.5%), and Germany (21.2%), while a smaller number of patients go to France (7.8%), Spain (3.2%), the Czech Republic (6.2%), and Slovakia (2.1%) [10]. Despite the weak existing local medical infrastructure and the heavy reliance on international healthcare systems for the treatment of citizens, investments in research in the health sciences is meager in the State of Kuwait [11]. Therefore, the state of Kuwait needs to introduce significant overhauls to its healthcare system, by upgrading the treatment facilities within the country to meet the local needs and avoid external reliance. Improving the efficacy of the relevant governing agencies and the efficiency with which they implement relevant policies is the most critical aspect of this process [12].

The Ministry of Health is the main governing body responsible for maintaining the health of the population, which is separated into six health regions—Capital, Hawalli, Ahmadi, Mubarak Al-Kabeer, Jahra, Farwaniya, and Al-Suabah. A health region is a decentralized administrative unit with significant autonomy, and is headed by a Director of Health. Primary healthcare is offered at the community level through polyclinics, while specialized care is offered through tertiary hospitals and some specialized hospitals, all of which are owned by the government. As of 2014, the total number of healthcare workers was 56,812; this included physicians (14%), nurses (37%), administrators (19%), medical technicians (16%), non-medical technicians (6%), dentists (3%), pharmacists (2%), and other service personnel (2%) [13]. The ratios of healthcare workers to the population served are as follows: 1.9 physicians, 0.4 dentists, 0.3 pharmacists, and 5 nurses per 1000 people. There were 2045 physicians, 813 dentists and 6348 nurses working in the private healthcare sector in 2015, and the proportion of healthcare workers was similar to the government sector [13].

The most vulnerable area within the state of Kuwait’s healthcare system is health information. This field of health information is closely related to the development of healthcare-related technologies, and to the development of the healthcare industry as a whole [10]. The primary healthcare clinics maintain electronic healthcare records (EHRs) of each patient, while only three hospitals have implemented EHRs at the secondary care level. EHRs include the discharge summary of the patients and the same data are sent to National Center for Health Information (Health & Vital Statistics Department) within the Ministry of Health. The data are sent back to the hospitals after manually checking the discharge summaries for any inaccuracies, which are corrected accordingly [14]. Although health data is collected in the state of Kuwait by various stakeholders, the Healthcare Information System has a limited impact on policy decisions because of its poor quality, relative inaccessibility, poor governance, and lack of proper usage. The Healthcare Information System may be strengthened by improving the quality of medical records reported to the Ministry of Health. A national health information management strategy is needed, to capitalize on existing health information system and data infrastructure and guide public health policies [10].

If the state of Kuwait wishes to increase investments into the healthcare system and into research in the health sciences, it is imperative that a translational research complex be established, along with an investor-friendly framework, to attract and encourage investors. Lack of local investments in the healthcare system and in medical technologies leads to increased reliance on foreign resources and labor, which in turn leads to increased distrust and decreased use of the local healthcare system, including receipt of treatments and medical care.

The main reasons for the decline in productivity in the fields of healthcare technology and research in the state of Kuwait are the lack of a national development plan for the healthcare industry, lack of translational research complexes associated with hospitals, and weak or non-existent national policies pertaining to pharmaceutical and health technology. The absence of hospitals that accommodate translational research activities and complexes in the State of Kuwait delays advancements in local healthcare informatics, and hinders overall research, development, and innovation in the healthcare industry.

## 2. Opportunities in Health Knowledge

Health knowledge is a crucial engine for the development of clean industries that are based on human resources and a stable economic base. Applying the latest relevant knowledge and skills obtained through research is the most effective way to influence the healthcare industry, and health knowledge is essential for carrying out impactful translational medical research [15].

Healthcare services and technologies have led to economic growth in many countries around the world. According to a report from the Kuwait Foundation for the Advancement of Sciences (KFAS) [8], healthcare-related fields offer promising areas for economic development in the State of Kuwait. In order to tap into this potential, the State of Kuwait needs to first invest in the establishment of a solid base of healthcare knowledge and technologies.

Key steps towards maximizing all the potential economic benefits from the healthcare sector include creating a national development plan for the healthcare industry, and establishing a translational medical research complex, in affiliation with a local hospital.

Translational research, also known as bench-to-bedside research, consists of the creation of new scientific knowledge, and its subsequent integration into clinical practice. Due to the complexity of current clinical practice, and the lack of availability of patient knowledge, the application of any newly derived knowledge is becoming increasingly challenging. Creative planning of the national health industry and translational research studies in the state of Kuwait will spur local healthcare technology innovation.

Health technology and medical tourism are among the components of national economic growth in many countries, including South Korea, India, Thailand, the United Kingdom, the United States, Germany, Singapore, and Japan [16,17,18]. In fact, Japan, Singapore, South Korea, the United Kingdom, and the United States have been investing in medical translational research complexes for a considerable period of time [16,17,18].

## 3. Impact of R&D in Healthcare-Related Fields on Economic Development

Improvements in healthcare services and infrastructure within a nation constitute an essential component when it comes to assessments of economic development, and are the building blocks for sustained economic growth [19]. Thus, while life expectancy figures show no significant correlation with the long-run growth rate at the steady state, life expectancy does positively affect long-run economic growth [19]. In fact, Prettner shows that increasing life expectancy has an unambiguously positive effect on technological progress and long-run economic growth, in the cases of both endogenous and semi-endogenous growth models [20]. According to the endogenous growth models, in the presence of adequate R&D investments, population size is the major determinant of a country’s long-term economic development [20]. The assumption is that the larger the number of scientists involved in R&D efforts within a country, the greater the country’s ability to innovate, generate profits, and create opportunities in these large markets. In contrast, semi-endogenous growth models are based on the idea that long-term economic performance is affected by population growth rather than population size [20]. Based on this model, the continuous production of new technologies needed to keep pace with the ever-increasing demand for technological progress becomes increasingly challenging, thus requiring the involvement of more scientists in R&D efforts. Therefore, positive population growth is thought to be essential for long-term economic development.

According to a recent paper published by the IZA—Institute of Labor Economics, “Increasing life expectancy results in higher aggregate savings, which puts downward pressure on the equilibrium interest rate. This, in turn, raises the discounted stream of income derived by investing in successful R&D projects. The incentive to carry out R&D increases, which boosts technological progress and long-run economic growth” [19].

The aggregate human capital stock, rather than the size of the workforce allocated to R&D, is what matters for long-run economic growth [21]. The complementarity between health, healthcare, and education is crucial when it comes to raising the human capital level and the intermediate input into the healthcare R&D sector [19]. Importantly, studies have shown that long-run economic growth rises with increased investments in the healthcare industry [19].

While scope exists for improving the efficiency of healthcare systems throughout, economic growth alone is a poor benchmark for valuing the desirability of health and healthcare [19]. Within developed economies, the benefits of even modest health improvements likely far outstrip losses in forgone consumption [22]. The increase in medical innovation spurred by the generous provision of healthcare compound these positive outcomes [19].

**Figure 1:**
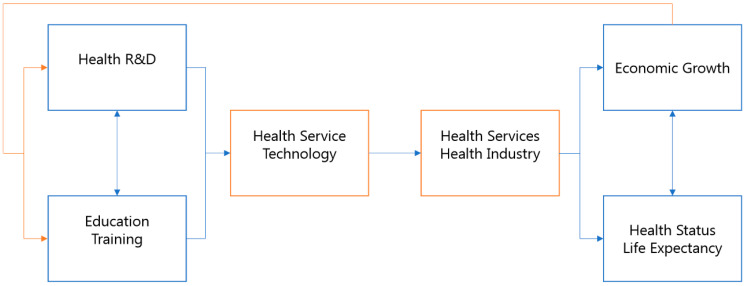
A virtuous cycle of local investments in R&D, improved health status among the local population, and national economic growth.

Overall, the expansion of investments into healthcare R&D leads to improvement of health service technologies, which not only improves the quality of health services, but also stimulates related industries. The improvement of healthcare services and the vitalization of the healthcare industry not only enhance the level of health of the general population, but also leads to national competitiveness and economic growth. The demand for skilled manpower will create a healthy competitive environment, in which students seek postsecondary education to earn higher degrees and increase their training and proficiency in various technological disciplines. This will lead to a new generation of researchers and a technologically skilled workforce, that will create the base for a sustainable future based on R&D-led innovation. The general population will also enjoy improved quality of life by virtue of the advanced health technologies that are introduced. In this process, there will be a qualitative and quantitative improvement of not only research personnel, but also individuals capable of interacting with and optimally utilizing advanced technologies, individuals who manufacture products related to the health industry, and healthcare service providers. This improved national health level and economic growth will lead to an increase in healthcare R&D and will create a virtuous cycle, as schematized in Figure 1.

The need for investment to create a knowledge-based economy is not a new concept—the idea of a knowledge-based economy emerged in the 1990s when European countries joined the race to catch up with the technological development in the United States of America [23,24]. The knowledge-based economy is defined as production and services based on knowledge-intensive activities that contribute to an accelerated pace of technical and scientific advancement [25]. Rapid expansion of knowledge-intensive industries in the recent years created new opportunities for economic growth, in spite of a steep global rise in competitiveness. Creation, transfer, and preservation of knowledge are the critical elements that a country needs in order to attain a sustainable position in today’s competitive world [26]. The healthcare industry in Kuwait was identified as one of the priority sectors in need for development taking into consideration many factors, including local demand, availability of resources, and prospects for its contribution to create a knowledge-based economy [8]. However, the key stakeholders in Kuwait must develop the necessary infrastructure needed to achieve this objective, as discussed further in the following sections.

## 4. Increase Investments in Healthcare-Related R&D Activities

As discussed earlier in the context of R&D spending and economic development, spending on R&D can be likened to building a market. Once the market is established, people gather to produce knowledge through research, with some individuals who want to process the produced knowledge, and some who want to buy processed knowledge. As the demand for better knowledge production grows, educational institutions begin to produce skilled researchers to meet that demand. Since markets generally encourage competition, they tend to tangentially encourage the production of better knowledge, better-skilled workers, and better products.

Kuwait has enough educational institutions, but the infrastructure for producing basic knowledge is very weak. Thus, there is an absolute shortage of researchers producing knowledge [27]. There is only one public university in the state of Kuwait—Kuwait University, which caters to the needs of higher education for the whole population. A few private universities were established very recently, which offer technical education without any significant research facilities. Kuwait Institute for Scientific Research (KISR) is the only institution other than Kuwait University which has modern research facilities within its four research centers (Petroleum, Water, Energy and Building, and Environment and Life Sciences). The KISR mandate dictates conducting applied research other than medicine and healthcare-related research, and therefore does not exert significant efforts towards basic research. There is a need for a place to produce knowledge, to process it, and to buy and sell it. While funds for forming a healthcare “market” in the state of Kuwait are available, a market cannot be formed with funds alone, and what is lacking at the moment are the funds to produce knowledge. Such funds can be attained through investments in R&D.

However, Kuwait’s R&D expenditure is low, 0.08% of GDP in 2016 [28]. The state of Kuwait should ensure that R&D investments will reach 0.2% of GDP in 2020, 0.4% in 2021, 0.6% in 2022, 0.8% in 2023 and 1.0% in 2024. It is desirable to continue to expand R&D investments to 2.5%, which is the average investment rate in high-income countries.

## 5. Design Investments in Healthcare-Related R&D Activities

Healthcare-related R&D activities in the State of Kuwait should be within the scope of the nation’s overall R&D investment design. Innovation is often achieved through interdisciplinary, rather than through the achievement of any one field by itself. For example, the latest medical technologies are completed through the convergence of nanochemistry and biology, biomedical engineering, and physics. Even if a country develops R&D infrastructure for a specific field, innovation must occur in an environment in which productivity in other related fields can simultaneously benefit. Although the medical sector is leading the way in the State of Kuwait when it comes to innovations, R&D investments must be designed in such a way that supports relevant healthcare-related scientific research as well.

This article explains how to design R&D in medical and health sciences, which involves several essential principles. The first principle is to have a comprehensive design that encompasses all aspects of healthcare and involves cutting-edge health research, including cancer research, cell biology, systems biology and medicine, population health science, biotechnology, pharmaceutical research, epidemiology, molecular and genomic medicine, bio-medicine and nano-medicine, environmental health science, immunology research, neuro-psychiatry research, and more.

The second principle is transparency, which entails making information and data publicly available. This includes every type of healthcare-related information, such as vital statistics, data in healthcare information systems, public health information systems, population-based surveys, patient’s satisfaction surveys, student health surveys, industrial and environment pollution data, international survey, and other health-related data and surveys. Exclusions and restrictions to public sharing of information can be applied to maintain privacy of sensitive patient data, as assessed by an ethical review board.

The third principle is to establish solid connections with both the local and global medical industries, which will promote health technology utilization, establish a foundation for growth, and increase global market access. The Kuwait Foundation for the Advancement of Sciences (KFAS) could play a leading role in achieving this goal. Other institutions with the capacity to bring new technologies to Kuwait for research and development include KISR, the Dasman Diabetes Institute, and Kuwait University.

The fourth principle is to encourage cross-sectoral integration and interdisciplinary collaboration. Doing so will allow for collaborative research projects between the national health research institutes and medical centers and hospitals. Meanwhile, establishing national health research institutes will provide opportunities for foreign scientists to conduct collaborative research locally. It is important to note that R&D design must be linked to national policies, including policies related to the healthcare industry, health research, health technology, and health information communication. Furthermore, integration of any data produced with existing national data and making all of the information publicly available is paramount for reaching the desired outcome. Although health data are recorded in an electronic format in primary healthcare centers and some major hospitals in Kuwait, the system is not sufficiently developed to readily assess disease burdens, due to the lack of an integrated Health Information System [10]. Periodic assessment of population health is a standard practice employed by most of the developed countries of the world. National Population Health Surveys should be conducted at five year intervals and data should be collected by using standard templates, with the help of an expert survey design team [10]. Encouraging data integration will ensure efficiency, consistency, regularity, reliability, and accuracy, especially in the context of population-based data. All of this is essential for assuring that evidence-based policy decisions are built on solid, comprehensive, and updated information.

The fifth principle involves taking into consideration capacity building for individuals and institutions. This can be achieved by supporting a variety of educational programs, ranging from specialist professional training for doctors to pre- and post-doctoral training and summer student research training for research scientists. Overall, these principles will achieve an impactful extension of R&D for the enhancement of healthcare services and technologies and R&D planning capabilities.

## 6. Develop a Healthcare R&D Management System

**Figure 2:**
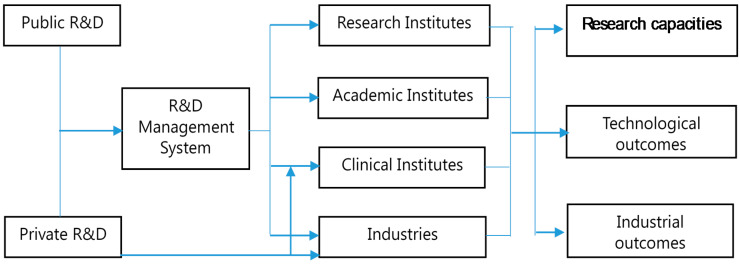
The paths of contribution of R&D in research-related institutes.

National R&D should be well-operated and based on well-designed national R&D plans and policies. A R&D management system will serve to determine R&D investment priorities, both in terms of investment areas and the size of investments. Such a system will also assist in the establishment of relevant impactful institutions that conduct R&D activities. Furthermore, a R&D management system will serve to organize and evaluate R&D performance, share results, and incorporate relevant results in the overall national development plan. The role of carrying out such essential tasks does not lie solely within the public sector, but can also involve the private sector. Thus, R&D management does not only involve management of funds, but also the management of R&D activities at both the institutional and national levels. Such activities will involve keeping track of R&D funds, research institutes, researchers, educational institutions, and industries. Therefore, the responsibility of performing R&D management is not at the institutional level. Rather, such a system must be managed by a governmental entity that can integrate R&D-related information from various fields, and incorporate the data to optimally design the national development plan for the country. Figure 2 shows how the R&D management system is connected to various institutions, and the outcomes of implementing such a system.

## 7. Strengthen Research Infrastructure

Efficient R&D operations require research institutes and researchers to lead research and development activities. In particular, research that specializes in healthcare requires a significant amount of collaboration across several disciplines. This is because research in the healthcare sector covers the entire life cycle of human life—promoting health, preventing and treating diseases, and rehabilitation. Furthermore, the outcomes of health sciences research and development are not aimed at individual preventive treatment alone. Rather, they are aimed at the community and national efforts together. Therefore, investments in the healthcare R&D sector need to encompass investments in several other relevant fields, in order to be maximally productive. Other fields that support healthcare-related research are biology, chemistry (particularly nanochemistry), information and communications technologies, and physical sciences. Additionally, depending on the overall purpose of promoting investments in healthcare-related R&D, as defined in the national development plan, various related academic fields may also need to receive additional support.

It is worth noting that investments in the following health-related R&D fields can be of immediate relevance to the state of Kuwait: stem cell research, genome science, biomedical science, laboratory control of infectious diseases, health information and communication technologies (ICT), diagnostic science, clinical nutrition, pharmaceutical science, physical science, and public health science. Capacity building for conducting research in these disciplines will help the state of Kuwait achieve its major development goals related to healthcare and economic growth, as stated in the Kuwait Vision 2035 Development Plan [1].

Researchers and institutions with research capabilities are needed to conduct these studies. In addition, the infrastructure should be strengthened, to ensure that these findings can be quickly commercialized for use in the clinic. Currently, institutions with the capacity to begin such research in Kuwait are the Kuwait Institute for Scientific Research and Kuwait University. Government hospitals can also carry out partial studies if certain conditions are met, however, their facilities are almost exclusively reserved for routine clinical diagnostic purposes. The most important point in health research is to study local medical needs, and to have the proper infrastructure needed to support research aimed at addressing such needs, as well as infrastructure capable of translating research findings to clinically applied materials. Such a comprehensive workflow can be carried out by translational research complexes, as discussed in the next section.

## 8. Establish an Industry-Based Translational Research Complex

The state of Kuwait should invest in translational research and in the establishment of a university teaching hospital, to enhance medical knowledge, innovation, and technological progress. Translational research is an interdisciplinary branch of the biomedical field, supported by three main pillars: benchside, bedside, and community. “Its goal is to combine disciplines, resources, expertise, and techniques…to promote enhancements in prevention, diagnosis, and therapies” [29]. The state of Kuwait lacks a research-based medical university, and Kuwait University does not have its own teaching hospital that focuses on “translational” medical research in which patient care can be informed by the most recent advances from the medical research frontier. The lack of such a translational research facility may act as a barrier towards achieving ‘quality education and knowledge development’ [30,31]. Furthermore, it hinders the development of healthcare knowledge and technologies, and undercuts the quality of medical education in the state of Kuwait. As a result, the state of Kuwait now finds itself dependent on foreign countries for the provision of many healthcare services, pharmaceutical products, and medical technologies.

These are fundamental ingredients for creating an international medical hub that can promote trust in the local medical infrastructure and attract international patients seeking medical treatment to the country, thereby promoting medical tourism in the country.

Furthermore, by building innovative medical hospitals that offer treatments and technologies on the medical frontier, the government of the state of Kuwait will likely be able to convince local patients to remain in the country for medical care, thereby reducing the costs associated with sending patients for treatment abroad.

A translational research complex combines research, diagnostic, and treatment expertise across disciplines. As a result, it rapidly facilitates the development and spread of relevant medical knowledge, skills, and technologies.

Translational research is an interdisciplinary branch of biomedicine, supported by three main pillars (bench-side, bedside, and industry) that are aimed at enhancing the prevention and diagnosis of diseases, and improving related therapies [29].

The main purpose of establishing global translational research complexes and medical hub policies are to develop creative and innovative health knowledge and technology services and tools that can facilitate achieving the status of best medical hub globally and promoting inbound medical tourism from around the world. By building innovative medical research hospitals and developing innovative treatments and technologies, the State of Kuwait can develop an international medical hub for outpatient care, thereby improving the international standing of the State of Kuwait. Furthermore, such a facility will involve increasing spending expenditures on recruiting medical tourists from other countries. Given these advantages, there is a priority to invest resources in the health science R&D sector.

Health science R&D investments will serve long-term goals for the development of the healthcare industry. The state of Kuwait may set the goal of establishing a hub for medical tourism that can attract medical tourists, not only from the Middle East but also from other regions. This is very consistent with the translational research complex plan. In order to achieve this feat, there are several issues that need to be taken into consideration.

First, mechanisms for carrying out translational health sciences research and bringing novel healthcare technologies to fruition need to be provided, to assure maximal benefits to relevant individuals. Research conducted and resultant technologies developed need to encompass prevention, diagnosis, and treatment of diseases, health promotion, and improvements in rehabilitation and long-term care.

Second, cover medical tourism market, medical hub, bio-health industry, pharmaceutical industry, medical instrument industry, healthy food industry, and beauty-cosmetic industry.

Third, strategies for the promotion of prospective medical equipment manufacturers (small- and medium-sized companies) and members of the pharmaceutical industry need to be developed. Other than healthcare products, potential developed technologies can include beauty products and nutritional products. Thus, measures for collecting information about relevant markets including overseas markets need to be established. Next, the translational research complex and medical hub need to be designed. Finally, a detailed implementation plan needs to be composed. The plan should include details pertaining to the operator of the translational research complex, the companies and research institutes involved, financial expenditures, facilities, workforce, etc.

## 9. Conclusions

Research and development expenditure in the state of Kuwait is low; 0.08% of GDP in 2016, and research institutes are rare. Given these conditions, it becomes increasingly difficult to retain stellar researchers who can facilitate innovation. Furthermore, these conditions have rendered investments in health science R&D activities meagre in the state of Kuwait. Insufficient investments in health science and medical science is one of the underlying causes of challenges that the healthcare system currently faces. This insufficient investment causes not only weak health knowledge, but also the weakness of health information, lack of health technology, and meagre health industrial development.

The health sciences field is one of the most promising areas for economic development in the State of Kuwait. The expansion of investment in health science R&D can lead to the improvement of health service technologies, which not only improves the quality of healthcare services, but can also serve to stimulate related industries. In this process, there will be a qualitative and quantitative improvement of not only research scientists, but also those who deal with advanced technologies, those who manufacture and those who provide health care services. Such changes can contribute to establishing a knowledge-based economy and to improving the overall health status in the state of Kuwait. In the era of the knowledge-based economy, innovation and sustainable economic growth require high-quality researchers to conduct research. When the market for research is activated, such advanced human capital is nurtured. Without a market, human capital is not cultivated.
